# Alzheimer’s Risk Gene TREM2 Determines Functional Properties of New Type of Human iPSC-Derived Microglia

**DOI:** 10.3389/fimmu.2020.617860

**Published:** 2021-02-03

**Authors:** Marvin Reich, Iñaki Paris, Martin Ebeling, Nadine Dahm, Christophe Schweitzer, Dieter Reinhardt, Roland Schmucki, Megana Prasad, Fabian Köchl, Marcel Leist, Sally A. Cowley, Jitao David Zhang, Christoph Patsch, Simon Gutbier, Markus Britschgi

**Affiliations:** ^1^ Roche Pharma Research and Early Development, Neuroscience and Rare Diseases Discovery and Translational Area, Roche Innovation Center Basel, F. Hoffmann-La Roche Ltd, Basel, Switzerland; ^2^ In Vitro Toxicology and Biomedicine, Department inaugurated by the Doerenkamp-Zbinden Foundation, University of Konstanz, Konstanz, Germany; ^3^ Achucarro Basque Center for Neuroscience, Science Park of the UPV/EHU, Leioa, Spain; ^4^ Roche Pharma Research and Early Development, Pharmaceutical Sciences, Roche Innovation Center Basel, F. Hoffmann-La Roche Ltd, Basel, Switzerland; ^5^ Roche Pharma Research and Early Development, Therapeutic Modalities, Roche Innovation Center Basel, F. Hoffmann-La Roche Ltd, Basel, Switzerland; ^6^ James Martin Stem Cell Facility, Sir William Dunn School of Pathology, University of Oxford, Oxford, United Kingdom

**Keywords:** iPSC (induced pluripotent stem cell), microglia, cell culture protocols, drug development, TREM2 (triggering receptor expressed on myeloid cells), Alzheimer’s disease (AD)

## Abstract

Microglia are key in the homeostatic well-being of the brain and microglial dysfunction has been implicated in neurodegenerative disorders such as Alzheimer’s disease (AD). Due to the many limitations to study microglia *in situ* or isolated for large scale drug discovery applications, there is a high need to develop robust and scalable human cellular models of microglia with reliable translatability to the disease. Here, we describe the generation of microglia-like cells from human induced pluripotent stem cells (iPSC) with distinct phenotypes for mechanistic studies in AD. We started out from an established differentiation protocol to generate primitive macrophage precursors mimicking the yolk sac ontogeny of microglia. Subsequently, we tested 36 differentiation conditions for the cells in monoculture where we exposed them to various combinations of media, morphogens, and extracellular matrices. The optimized protocol generated robustly ramified cells expressing key microglial markers. Bulk mRNA sequencing expression profiles revealed that compared to cells obtained in co-culture with neurons, microglia-like cells derived from a monoculture condition upregulate mRNA levels for Triggering Receptor Expressed On Myeloid Cells 2 (TREM2), which is reminiscent to the previously described disease-associated microglia. TREM2 is a risk gene for AD and an important regulator of microglia. The regulatory function of TREM2 in these cells was confirmed by comparing wild type with isogenic TREM2 knock-out iPSC microglia. The TREM2-deficient cells presented with stronger increase in free cytosolic calcium upon stimulation with ATP and ADP, as well as stronger migration towards complement C5a, compared to TREM2 expressing cells. The functional differences were associated with gene expression modulation of key regulators of microglia. In conclusion, we have established and validated a work stream to generate functional human iPSC-derived microglia-like cells by applying a directed and neuronal co-culture independent differentiation towards functional phenotypes in the context of AD. These cells can now be applied to study AD-related disease settings and to perform compound screening and testing for drug discovery.

## Introduction

Microglia play a key role in the well-being of the brain by fulfilling various functions in development, homeostasis and the first-line immune defense (1–7). Alzheimer’s disease (AD) is a devastating age-related neurodegenerative disorder where microglia have been implicated for over a century in the pathogenesis based on neuropathological findings and by mimicking microglia dysfunction in preclinical models (8). More recently, genome wide association studies substantiated the long-time proposed active implication of microglia within initiation and progression of AD and other neurodegenerative diseases of the central nervous system (9, 10). Together, this strongly supports the rationale for developing therapies that pharmacologically modulate microglia.

In order to facilitate investigating the biology of microglia and to make them available for drug screening assays, cellular models had to be established. Until recently, *in vitro* studies with microglia have been limited to either employing primary rodent cells or cell lines (e.g. BV2) (11). Due to the stress implicated during their isolation process and the loss of tissue context, primary cells rapidly alter their previous *in situ* microglial properties (12, 13), and batch-to-batch variations as well as impurities are known hurdles of this approach. Moreover, generation of primary cells requires either euthanizing large numbers of animals or accessing difficult to obtain highly characterized human brain samples with short postmortem delay. Both approaches result in only a small number of cells, which in turn limits the throughput for compound screening campaigns or larger biological studies (14, 15). In contrast, due to their proliferative nature, cell lines do not have limitations in cell numbers, can be of human origin and are therefore often used in screening setups (16). However, due to their immortalization or neoplastic-origin, cell lines show strong discrepancies compared to the desired *in vivo* characteristics (17, 18).

With the arrival of human induced pluripotent stem cell (iPSC) technology (19, 20), and with the evolving understanding of microglial origin (21–23), several methods were reported for the generation of iPSC-derived microglia-like cells (24–26), hereafter called iPSC microglia. These protocols commonly aim to resemble the yolk sac ontogeny for the generation of primitive macrophage progenitors. Current reports indicate that iPSC microglia seem to be superior to primary cells or cell lines with regard to expressing key microglial marker genes (25). Importantly, unlike primary cells, iPSC microglia or their macrophage precursors can be generated robustly and in a controlled manner in scalable amounts (25, 27). This makes iPSC microglia ideal for drug screening and for extensively studying biological mechanisms under conditions resembling better the physiological state of microglia. Additionally, iPSC based models provide the opportunity to study the effect of disease associated genes with isogenic mutations or knockout pairs.

Despite the advances in developing cell culture models, *in vitro* microglia often lack important properties such as modulating the expression of a fully functional repertoire of various surface receptors, which microglia require to interact with their environment (28). For instance, microglia are the major cell type in the brain to express Triggering Receptor Expressed On Myeloid Cells 2 (TREM2). Signaling through this receptor modulates crucial microglia functions such as phagocytosis, proliferation, survival, and lipid metabolism in homeostatic, inflammatory or neurodegenerative conditions [extensively reviewed in (29, 30)]. Mutations in TREM2 are associated with an increased risk to develop various neurodegenerative disorders including AD (9, 10, 31–36). In the context of amyloid plaques, a neuropathological hallmark of AD, TREM2 was found in preclinical experiments to be essential for the metabolic fitness and transition of homeostatic to disease-associated microglia (DAM) (37, 38). Although the function and role of TREM2 in AD pathogenesis remains unclear, it became a key target for potential therapeutic intervention (39–41).

Some TREM2 loss-of-function-related phenotypes in the context of AD were recently described by others in iPSC-derived microglia (42–45). While the literature about iPSC microglia is growing, more descriptions are needed to compare different approaches that generate robust and scalable human cellular models of microglia. Such cellular models of microglia will hopefully become soon more translatable to microglia in the brain thereby establishing themselves as valuable tools to study disease mechanisms and to perform compound screens for drug development *in vitro*.

Here, we present the optimization of a protocol to generate iPSC microglia in a monoculture condition and explored whether these cells can serve as a model to study microglia function and gene expression in the context of TREM2 modulation. Building on our previously published large scale differentiation protocol of myeloid progenitors from iPSC (27), we have extended the differentiation of these myeloid progenitors for additional 14 days to microglia-like-cells. As a major distinction from previously published co-culture methods, we observed in iPSC microglia from monoculture an increased TREM2 mRNA expression. The regulatory function of TREM2 in these cells was confirmed by comparing wild type with isogenic TREM2 knock-out iPSC microglia. The overall approach resulted in a work stream to generate human iPSC microglia by a directed and neuronal co-culture independent differentiation resulting in distinct phenotypes for mechanistic studies in AD. Our iPSC microglia protocol can now be applied to scale up the production of these cells, study certain AD-related disease settings, and perform compound screening and mechanistic experiments in drug development.

## Materials and Method

### iPSC Culture

All work with human iPSC and the derived cell types was performed under the respective Swiss legislation, ethical guidelines, and approval. All media compositions are summarized in **Table S1**. We recently reported an improved and highly scalable variant of the method published by van Wilgenburg et al. (27, 46) for the differentiation of iPSC to primitive macrophages. In brief, for iPSC maintenance culture dishes (Corning) were coated with 12.5 µg/ml rhLaminin-521 (BioLamina). Human iPSC were seeded and cultured in mTesR1 media (StemCell Technologies) at 37°C with 5% CO_2_ and media was changed daily. Cells were passaged at 90% confluency, media was removed, cells were washed 1x with PBS and detached with Accutase™ (Innovative Cell Technologies) for 2 to 5 min at 37°C. After removal of Accutase™ by centrifugation cells were either used for maintenance or start of differentiation. The cell lines used in this study, Bioneer WT (BIONi010-C) and Bioneer C17 (BIONi010-C17/TREM2 KO) were obtained from Bioneer. Cells were quality controlled by STR profiling, SNP phenotyping and karyotyping after banking. To avoid genetic drift and variations sub-culturing was limited to an absolute minimum.

### Differentiation of iPSC to Primitive Macrophages

#### Embryoid Body (EB) Generation

This step was performed as previously described (27). Briefly, to obtain uniform EBs, iPSCs were seeded into AggreWell 800 (StemCell Technologies) plates. Two ml mTesR1, supplemented with 10 μM ROCK inhibitor (Y27632, StemCell Technologies) and containing a single cell suspension of 4*10^6^ iPSCs were added to each AggreWell and centrifuged for 3 min at 100xg to assure an even and fast distribution of the iPSC into the AggreWells. The next day, mesoderm and subsequent hemogenic endothelium induction was started by exchange of 75% of the mTeSR1 media with mTeSR1 media supplemented with 50 ng/ml rhBMP4 (biotechne), 50 ng/ml rhVEGF (biotechne), and 20 ng/ml rhSCF (biotechne), and repeated the following 2 days.

#### Plating of EBs and Continued Maturation Along the Myeloid Lineage

On day 4 of differentiation, EBs were harvested and transferred to factory media, consisting of X-VIVO 15 media (Lonza) supplemented with 2 mM GlutaMAX (Thermo Fisher Scientific), 10 U/ml Penicillin/Streptomycin (Thermo Fisher Scientific), 50 µg/ml Mercaptoethanol (Thermo Fisher Scientific), 100 ng/ml rhM-CSF (Miltenyi Biotech), and 25 ng/ml rhIL3 (Miltenyi Biotech). EBs were plated at a density of 1 EB/cm^2^ in growth factor reduced matrigel (Corning) precoated cell culture vessels and myeloid factories were matured as described previously (25).

#### Macrophage Progenitor Harvesting

**Figure 1:**
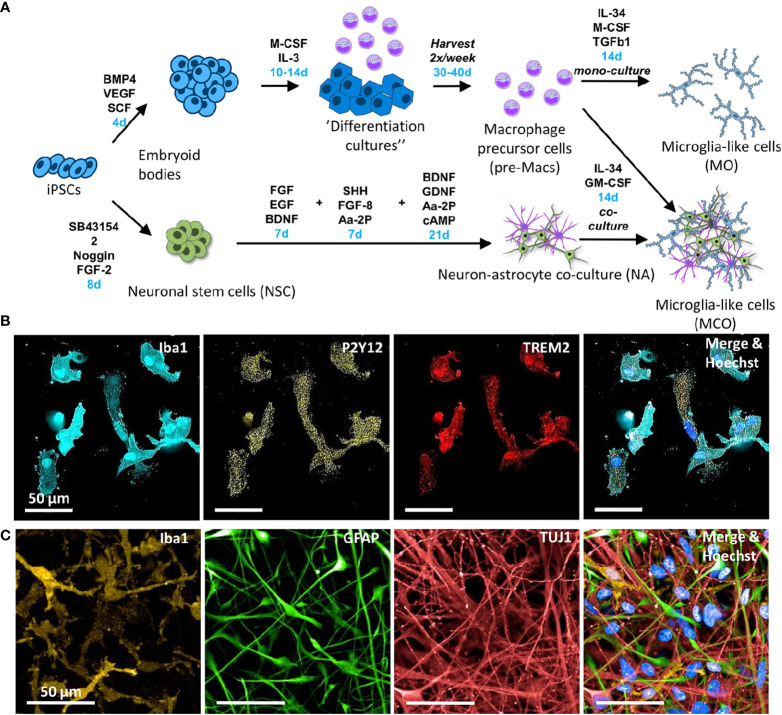
Approaches employed to differentiate human iPSC towards microglia-like cells. **(A)** Schematic diagram of the protocols to generate different microglia-like cells from human iPSC. Relevant growth factors and durations (blue) of differentiation steps are indicated. The upper part depicts the differentiation of iPSCs into “myeloid factories” that produce macrophage precursor cells (pre-Macs) (27). For the generation of monocultured iPSC-derived microglia, pre-Macs were differentiated in the presence of IL-34, M-CSF, and TGF-β1. The lower part depicts the differentiation steps from iPSCs to neural stem cells and further to neurons. For the generation of neural co-culture-derived microglia, pre-Macs were seeded on top of the neuron/astrocyte cultures. **(B)** Representative images of immunostaining of iPSC microglia after 24 days differentiation in monoculture. Monoculture-derived microglia-like cells stain positive for Iba1 (cyan), P2Y12 (yellow), and TREM2 (red). Cellular nuclei were labeled with Hoechst 33342 (blue). **(C)** Immunostaining of microglia-like cells, neurons and astrocytes after 14 days differentiation in co-culture. Representative images are shown from three biological replicates. Microglia-like cells are detected by Iba1 (orange), astrocytes by GFAP (green), and neurons by TuJ1 (red), respectively. Cellular nuclei were labeled with Hoechst 33342 (blue).

Macrophage progenitors were collected from the supernatant by centrifugation (4 min, 300xg) and were either matured into co-culture or monoculture microglia-like cells (**Figure 1A**).

### Differentiation of iPSC Into Microglia-Like Cells in Co-Culture

Induced pluripotent stem cells were differentiated to neurons using a protocol that was previously published (47). Neurons were quickly thawed at 37°C and seeded at a density of 200,000 cells/cm^2^ in neuronal differentiation media [consisting of 1:1 Advanced DMEM/F12 media (with GlutaMAX I) (Thermo Fisher Scientific) and Neurobasal media (Thermo Fisher Scientific) + 1% B27 supplement without vitamin A (Thermo Fisher Scientific), 1% N2 supplement (Thermo Fisher Scientific), 50 µg/ml Beta-Mercaptoethanol (Thermo Fisher Scientific), 10 U/ml Penicillin-Streptomycin (Thermo Fisher Scientific), 20 ng/ml rhBDNF (PeproTech), 10 ng/ml rhGDNF (PeproTech), 100 µM Aa2-P (Sigma Aldrich), 500 µM cAMP (BIOLOG Life Science), and 1 µg/ml murine laminin (Roche), supplemented with 10 µM ROCKi (Y27632, StemCell Technologies) for seeding]. After a 100% media change one day after seeding, 50% of the media was changed twice a week. After 14 days, macrophage precursor cells were added at a density of 160,000 cells/cm^2^. Therefore, the media was replaced with a macrophage precursor cell suspension in N2 media [Advanced DMEM/F12 + 1% N2 supplement, 10 U/ml Penicillin-Streptomycin, 2 mM Glutamax, 50 µg/ml β-ME, 100 ng/ml rhIL34 (Miltenyi Biotech), and 10 ng/ml rhGM-CSF (biotechne)]. Half of the media was changed twice a week for two additional weeks.

### Differentiation of iPSC to Microglia-Like Cells in Monoculture

Initially, different variations of coating and media were tested (indicated in figure legends). For the final protocol flasks and plates were coated with fibronectin. Fibronectin (Corning, 10 µg/ml in PBS^-/-^) was added and incubated for 3 h at RT, before washing three times with water. Macrophage precursors were seeded in RPMI media [RPMI 1640 media (Thermo Fisher Scientific) + 10 U/ml P/S supplemented with 100 ng/ml rhIL34, 25 ng/ml rhM-CSF, and 50 ng/ml rhTGF-β1 (PeproTech) at a density of 160,000 cells/cm^2^]. Half the media was changed twice a week for 14 days. On day 14, cells were replated by collecting cells from the supernatant and detaching adherent cells with Accutase™. These cells were replated into fibronectin pre-coated assay plates and cultured for at least two additional days prior to the assays.

### Magnetic-Activated Cell Sorting (MACS)

For the separation of co-cultured iPSC-derived microglia-like cells from neurons and astrocytes, immunomagnetic separation was applied. Cells were detached after 14 days in co-culture by incubation with accutase at 37°C for 45 min. After centrifugation at 300xg for 5 min, the cells were resuspended in N2 media containing 100 U/ml DNaseI (Roche) and incubated at RT for 10 min to minimize the amount of free-floating DNA and cell aggregates. Then, the cell suspension was filtered through a 70 µm cell strainer (Greiner). Magnetic labeling and magnetic separation using the autoMACSpro (Miltenyi Biotec) was performed using anti-CD45 MicroBeads (Miltenyi Biotech), following the CD45 MicroBeads separation manual, provided by Miltenyi Biotech. Higher specificity of antigen-binding was achieved by the addition of 12.5 µg/ml Fc Block (BD Biosciences) during incubation with the MicroBeads. Microglia-like cells were obtained in the positive selection.

### Quantitative Real-Time PCR

Cells were lysed and the RNA purified using the High Pure RNA Isolation Kit from Roche following the provided protocol. Macrophage precursor cell aggregates were lysed directly after the harvest, microglia-like cells, derived using the monoculture protocol were lysed directly in the cell culture plate. Co-cultured microglia-like cells were lysed directly after MACS.

For a one-step reverse transcription and PCR, the AgPath-ID One-Step RT-PCR kit (Thermo Fisher) was used. It contains an enzyme mix of reverse transcriptase and DNA polymerase. The reaction mixture was prepared according to the manufacturers descriptions and reverse transcription as well as PCR performed in LightCycler 480 384-well plates in a LightCycler 480 II (Roche) (reverse transcription for 10 min at 45°C, reverse transcriptase inactivation and initial denaturation for 10 min at 95°C, 50 cycles of 15 s denaturation at 95°C and 60 s annealing at 60°C). PPIA was used as a housekeeping gene and was detected simultaneously with the gene of interest using two different dyes (VIC for PPIA, FAM for the gene of interest). Specificity of the readout was ensured using no-enzyme and no-primer controls. A detailed list of the primers used can be found in **Table S2**.

### RNAseq and Data Analysis

#### Characterization by Bulk RNA Sequencing

Induced pluripotent stem cell derived macrophage progenitors, co- and monoculture microglia were generated as described above. Co-cultured microglia were purified as described above. All cultures were started at five different days to obtain five independent replicates for the RNAseq experiment. Cells were lysed and the RNA purified as described above. RNA purity was assessed using the Agilent 2100 Bioanalyzer. Strand-specific mRNA-seq libraries were generated from 1 µg total RNA using the TruSeq Stranded mRNA library prep kit (Illumina) according to manufacturer’s instructions. Briefly, mRNA was purified from total RNA by polyA capture, fragmented and subjected to first-strand cDNA synthesis. The second-strand synthesis was performed incorporating dUTP instead of dTTP to ensure strand-specificity. Barcoded DNA adapters were ligated to both ends of the double-stranded cDNA and subjected to PCR amplification. The resulting libraries were checked on an AATI Fragment Analyzer, quantified with Qubit and pooled. The resulting library pool was diluted for cluster generation on the cBot2 and finally sequenced on the Illumina HiSeq 4000 platform.

#### RNAseq Analysis

Base calling was performed with BCL to FASTQ file converter bcl2fastq v2.17.1.14 from Illumina (https://support.illumina.com/downloads.html). In order to estimate gene expression levels, paired-end RNASeq reads were mapped to the human genome (build hg38) with STAR aligner version 2.5.2a using default mapping parameters (48). Aligned reads were quality checked with FastQC and MultiQC version 1.7 (49, 50). Numbers of mapped reads for all RefSeq transcript variants of a gene (counts) were combined into a single value by using SAMTOOLS software (51) and normalized as rpkms (number of mapped reads per kilobase transcript per million sequenced reads (52),. RNA-seq data have been deposited in Gene Expression Omnibus (GEO accession number GSE159108).

#### Principal Component Analysis and Heatmaps

Principal component analysis (PCA) of the gene expression profiles was generated using ClustVis (53). Each dot in the PCA plot is a biological replicate. Heatmaps were generated using ClustVis and default settings (53).

#### Generation of MA Plots

To visualize changes in gene expression between different conditions MA plots were generated. The MA plots are based on gene expression levels measured in log_2_(RPKM), the logarithm to the base of two of the reads per kilobase of transcript per million reads sequenced. The x axis shows, for every gene, the average expression value between the two conditions that were compared. On the y axis, the difference between the two expression levels for every gene is depicted. Each gene is represented by a single dot. Some strongly affected genes were highlighted in yellow, with gene names specified.

#### Microglia Expression Modules

Microglia expression modules were derived from the publication of Friedman et al. (54) and complemented by two modules (DAM signatures TREM2 dependent and TREM2 independent) derived from the publication of Keeren shaul et al. (38). Gene list for expression modules can be found in **Table S3**. Differences between two groups in these expression modules were visualized in a Radar plot using python.

#### Gene Ontology Analysis

A gene ontology analysis was performed that used the differentially expressed genes that showed at least four RPKM difference between WT and TREM2 KO. The GO terms were condensed using the GO slim immune response tool from dice tools (55).

### Immunofluorescence Staining

Cells were fixed by replacing the medium with 4% PFA (Thermo Fisher Scientific, in PBS) followed by incubation at RT for 15 min. After washing three times using PBS, the cells were permeabilized by incubation in 0.1% PBS-T [0.1% Triton-X-100 (Sigma Aldrich) in PBS] for 15 min at RT. Following another washing step, non-specific binding sites were blocked by incubation in SuperBlock (Thermo Fisher Scientific) for 60 min at RT. The primary antibody was added in SuperBlock and incubated overnight at 4°C. Iba1, TREM2 and P2Y12 were added together, as well as Iba1, TuJ1, and GFAP. For the no-primary antibody controls, only SuperBlock was added. Following three washing steps, the cells were incubated with the three respective secondary antibodies (donkey anti-goat AF555, donkey anti-mouse AF647 and donkey anti-rabbit AF488, in SuperBlock) for 90 min at RT in the dark and unbound antibodies removed in two washing steps. Nuclei were counterstained with Hoechst33342 (Invitrogen, in SuperBlock) in the dark at RT, followed by two washing steps. The cells were kept in PBS and images acquired using the 63x objective of an OperaPhenix (Perkin Elmer). Images were processed and analyzed using the built-in Harmony software. The no-primary antibody control was used for background correction. A complete list of the antibodies used can be found in **Table S4**.

### Mitochondrial Respiration Assay

The assay was performed using the Seahorse XF Cell Mito Stress Test kit (Agilent). Cells were differentiated as described above (14 days monoculture microglia like) and, in a Seahorse XF96 cell culture microplate, 50,000 cells (480,000 cells/cm^2^) were seeded two days prior to the experiment. On the day of the experiment, the medium was replaced with 180 µl media for the mitochondrial respiration stress test (base medium + 2 mM L-glutamine (Thermo Fisher Scientific), 1 mM sodium pyruvate (Thermo Fisher Scientific), and 0.45% glucose (Sigma Aldrich), pH 7.4). Prior to the experiment, the cell culture microplate was incubated for 1 h at 37 ˚C in a non-CO_2_ incubator. The assay was performed using a Seahorse XFe 96 Analyzer and compounds (prepared according to the assay manual) were injected sequentially (1 µM oligomycin, 2 µM FCCP, and 500 mM rotenone/antimycin A) and the oxygen consumption rate (OCR) measured three times before treatment and after every injection. Data was processed and analyzed using the Seahorse Wave software. For normalization, cells were fixed with 4% PFA at 37°C for 15 min and nuclei stained with Hoechst 33342 for 15 min, before washing twice with PBS. The Operetta CLS high-content screening system (Perkin Elmer) was used for imaging nuclei counted and the ratio between cell types calculated.

### Transwell Migration Assay

Cells were differentiated as described above (14 days monoculture microglia like), detached from the dishes with accutase and plated at a density of 8,000 cells per well in a 96-well IncuCyte ClearView cell migration plate (Essen BioScience). In the lower compartment, either recombinant human complement C5a (biotechne, 1 ng/ml) or solvent control were added as chemoattractant, which generates by natural diffusion a gradient of the chemoattractant. Plates were incubated in an IncuCyte S3 (Essen BioScience) and images acquired using the 10x objective every 4 h for upper and lower well. Migration was assessed for 72 h and quantified using the Incucyte software Migration Analysis tool.

### Determination of Free Intracellular Ca^2+^

Cells were differentiated as described above (14 days monoculture microglia like) and 8,000 cells were plated per well of a 384 well plate. For calcium measurements, cells were incubated with the FLIPR calcium 6 imaging dye (Molecular Devices) following the manufacturer’s instructions. Briefly, dye was dissolved in 10 ml of assay buffer 1 and 20 µl per well were added to the cells. Cells were incubated for 2 h with the dye. Increase in cytosolic calcium in response to ATP (Thermo Fisher Scientific), ADP (Sigma Aldrich), as well as C5a was assessed using the Hamamatsu FDSS7000 detection system. Background signal (average of 10 pictures prior to addition of stimuli) was subtracted from the measured maximum following stimulation. RFU values were assessed per well.

### Phagocytosis Assay

Cells were differentiated as described above (14 days monoculture microglia like) and 40,000 cells were plated per well of a 96 well plate (Falcon 353219). For the phagocytosis assay, cells were incubated with Abeta coated, pHrodo labeled beads. To obtain these beads Amidine Latex Beads (A37322/Thermo Fisher Scientific) were washed once with PBS, pelleted by centrifugation (16,000 g/5 min) and incubated at 37°C overnight in PBS containing 1 mg/ml Aβ42 (AnaSpec). After incubation with Aβ42 beads were pelleted, washed with PBS and re-suspended PBS containing 0.2 mg/ml pHrodo™ Red, succinimidyl ester (pHrodo™ Red, SE/Thermo Fisher Scientific). Beads and pHrodo were incubated for 1 h at room temperature. After the incubation, beads were washed with PBS and re-suspended in PBS. Phagocytosis of beads was monitored using Incucyte S3 acquiring brightfield and red fluorescence images. Cells were recognized using the Incucyte software adherent cell-by-cell classification tool.

### Statistics

Unless otherwise mentioned, all data values are expressed as means ± standard deviation (SD). Unless otherwise indicated, experiments were performed at least three times (i.e., using three different cell preparations), with at least three technical replicates per condition. Statistical methods for analyzing the various data sets are indicated directly in the figure legends, data were analyzed using Graphpad Prism software.

## Results

For the generation of iPSC microglia many protocols still mostly rely on co-culturing microglia with neurons, astrocytes or neurons and astrocytes to mimic the brain environment (25, 26, 56). However, for drug screening purposes, e.g., with functional cellular assays, pure cultures of monolayer cells are highly desirable. One key aspect is to achieve proper microglia morphology *in vitro* in order to establish their functional phenotype as well (8).

In our hands, using the media condition that was closest to the previously published approach to generate iPSC microglia in monoculture (25), the cells showed only little ramifications (**Figure S1** N2, IL34, GM-CSF, Fibronectin). To increase ramification and marker gene expression of iPSC monoculture microglia, we began to screen existing protocols and tested six different media conditions (either N2 or RPMI media supplemented with either IL34+GM-CSF, IL34+M-CSF+TGF-β1 or IL34+M-CSF+TGF-β+CD200+CX3CL1) in combination with six different matrix coatings (Tissue culture treated only, Poly-D-lysine, CollagenI, Gelatin, Fibronectin, or Laminin) (**Figure S1A**). Conditions, where cells displayed ramifications, were chosen for follow up qPCR analysis (**Figure S1B**). Strongest differences between the different cultures were observed for key regulatory receptors of microglia such as fractalkine receptor CX3CR1, ADP chemoreceptor P2Y12, receptor tyrosine kinase AXL and lipoprotein lipase LPL mRNA expression. Based on the morphology and only minor differences upon CD200 and CX3CL1 addition for the last three days of differentiation, we chose for further analysis the condition of RPMI supplemented with IL-34, M-CSF and TGF-β1 on fibronectin coating (**Figure S1A** and **Figure 1A** for timeline). Under this condition, we confirmed the expression of P2Y12 on protein level by immunocytochemistry as well (**Figure 1B**). The iPSC microglia from monoculture displayed a similar morphology as the co-culture iPSC microglia (**Figure 1C**) suggesting that the fibronectin matrix resembles aspects of the matrix found in co-cultures.

**Figure 2:**
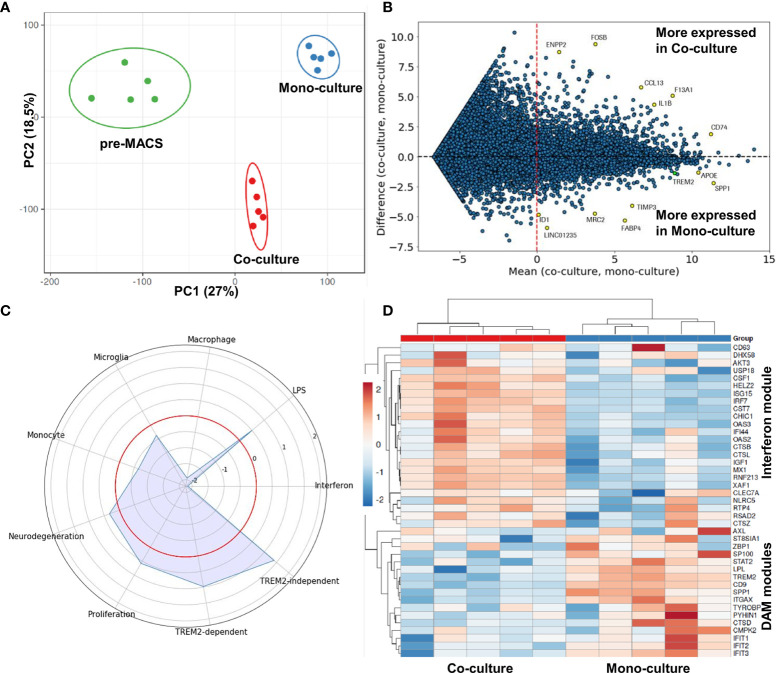
Comparison of gene expression profiles in mono- and co-culture-derived microglia. **(A)** Principal component analysis of complete expression profiles of macrophage precursors (pre-MACS), mono- and co-cultured microglia-like cells. The lack of any intersection between the ellipses (95% confidence interval) indicates a clear statistically significant separation between the gene patterns of the different cell types. **(B)** MA plot to visualize gene expression differences between mono- and co-culture-derived microglia-like cells. The MA plot is based on gene expression levels measured in log_2_(rpkm), the logarithm to the base of two of the reads per kilobase of transcript per million reads sequenced. The x-axis shows, for every gene, the average expression value between the two conditions, on the y-axis is, for every gene, the difference between the two expression levels. Some strongly affected genes are highlighted in yellow, with gene names specified. **(C)** Radar plot to visualize changes in gene expression in microglial modules. The red circle indicates the reference level as detected in co-cultured microglia. Peaks outside the red circle indicate higher expression in monoculture microglia and modules closer to the center indicate higher expression in microglia generated in co-culture with neurons. **(D)** Heatmap of the genes belonging to the DAM or interferon module in co- and monoculture derived microglia. Expression values were standardized and depicted on a z-scale with red indicating high and blue low expression, respectively. The five red and five blue boxes on top of the heatmap indicate the two different culture conditions, respectively, and that each culture condition was performed in five independent experiments. The downregulation of the “interferon” module and the upregulation of “DAM” modules is consistent between different experiments as indicated by the similar gene expression patterns between different biological replicates. This confirms the robustness of our new protocol.

To benchmark the microglia monoculture model towards iPSC microglia in co-culture with iPSC-derived neurons, we performed magnetic-activated cell sorting to remove the neurons and compared the microglia based on their bulk RNA-seq profile. A principal component analysis (PCA) demonstrated a sufficient cellular differentiation of the mono- and co-culture derived microglia with their common macrophage precursor (pre-Macs) (**Figure 2A**). Similarly, gene expression profiling between mono- versus co-cultured microglia revealed a higher expression of ENPP2, FOSB,CCL13, F13A1, IL1B, CD74 in microglia co-cultured with iPSC-derived neurons, whereas monocultured microglia expressed higher levels of ID1, LINC01235, MRC2, FABP4, TIMP3, APOE, and SPP1 (**Figure 2B**). Together, this confirms that the mono- and co-culture conditions induce two different microglia subsets.

Microglia are highly plastic cells that can change their morphological and functional phenotype as a reaction to different stimuli (8). Such stimuli can derive from environmental alterations in the brain due to aging and neurodegeneration. In support of this, comprehensive RNA-seq analyses of microglia isolated from human and mouse brain in an AD or other neurodegenerative disease context indicate an association between gene transcription pattern and a specific activation state of microglia. Recently, different transcription patterns were proposed to categorize microglia into different subsets (38, 54, 57, 58). In that context the upregulation of APOE and SPP1 as observed in our monoculture condition is part of a DAM signature (38). This indicates that in contrast to the co-culture condition, our monoculture condition provides stimuli that drive iPSC microglia towards a more disease associated expression pattern.

In order to explore the transcriptional pattern of the monoculture microglia further and to gain more insight into the different stimuli that possibly induced the transcriptional phenotype, we defined nine different modules of microglia states based on published data sets (See **Table S3** for the names of the genes and of the different modules) (38, 54). We then mapped the differences in gene expression profiles of the two culture conditions towards these modules (**Figure 2C**). Our mono- and co-culture iPSC microglia were similar in the “microglia” module validating the chosen differentiation conditions to generate microglia. The most prominent differences were observed for “macrophage” and “interferon” modules, which were lower expressed in the monocultured iPSC microglia. The reduced “interferon” signature is most likely attributed to the change from GM-CSF supplementation in co-culture to M-CSF supplementation in monoculture (59). The lower “macrophage” signature combined with the also slightly reduced “monocyte” module indicates an even stronger differentiation away from the peripheral myeloid cells than the co-cultured microglia. Furthermore, we observed upregulation of “TREM2-dependent” and “TREM2-independent” modules suggesting a more DAM-like phenotype for iPSC microglia in monoculture compared with microglia from co-culture. The downregulation of the “interferon” module and the upregulation of “DAM” modules is consistent between different experiments as indicated by the similar gene expression patterns between different biological replicates (**Figure 2D**).

**Figure 3:**
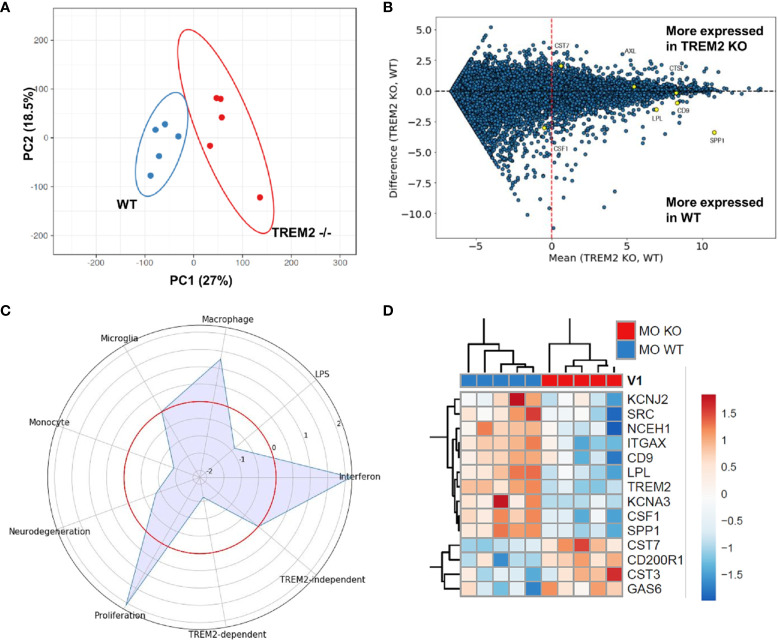
Differences in gene expression in wild type and TREM2 KO monoculture-derived iPSC microglia. **(A)** Principal component analysis of complete expression profile of monoculture derived microglia-like cells generated from wild type (WT) and TREM2 knockout (KO) iPSC lines. The lack of intersection between the ellipses (95% confidence interval) indicate a statistically significant separation between the gene patterns of the two cell types while the fact that the ellipses seem to touch each other indicate only a minimal separation in the PCA. **(B)** MA plot to visualize gene expression differences between WT and TREM2 KO monoculture iPSC microglia. The MA plot is based on gene expression levels measured in log_2_(rpkm), the logarithm to the base of two of the reads per kilobase of transcript per million reads sequenced. The x-axis shows, for every gene, the average expression value between the two conditions, on the y-axis is, for every gene, the difference between the two expression levels. Genes highlighted in yellow are part of the TREM2-dependent DAM signature genes according to Keren-Shaul et al. (38). **(C)** Radar plot to visualize changes in gene expression in microglial modules. The red circle indicates the reference level as detected in WT iPSC microglia. Peaks outside the red circle indicate higher expression in TREM2 KO microglia and modules closer to the center indicate higher expression in microglia generated from the WT iPSC line. **(D)** Heatmap of the genes belonging to the DAM module in WT and KO monoculture microglia. Expression values were standardized and depicted as z-scores with red indicating high and blue low expression, respectively. The five red and five blue boxes on top of the heatmap indicate the two different cell types, respectively, and that each cell type was analyzed in five independent experiments. The gene expression patterns for each cell type appear consistent between different experiments. This confirms the robustness of our new protocol for WT and TREM2 KO iPSC microglia.

At this point, we wanted to complement the DAM-like gene expression pattern of our monoculture iPSC microglia with functional data. Given the implication of TREM2 in the DAM-like expression pattern and the association of TREM2 with AD we focused on modulating this gene. To that end, we employed the isogenic TREM2 knock-out (KO) of the same iPSC line and differentiated the two cell lines in parallel in the same monoculture condition. As before, we first made sure there is an effect of TREM2 KO on a gene expression level. The PCA revealed a minor but clear separation in RNAseq-based gene expression between iPSC-derived microglia and their isogenic KO form (**Figure 3A**). The majority of differentially regulated genes were downregulated in the KO compared to the cells having functional TREM2 (**Figure 3B**). This included DAM-signature associated genes and became evident in the module analysis as well (**Figure 3C**). The iPSC-derived microglia lacking TREM2 also displayed an upregulation of the interferon, proliferation and macrophage module, while the monocyte and neurodegeneration module were downregulated. Confirming the genotype, TREM2 independent DAM signature stayed unaffected by the loss of TREM2. The upregulation of the “interferon” module and the downregulation of “DAM” modules in the TREM2 KO microglia compared with wild type cells is consistent and robust between different experiments as indicated by the similar gene expression patterns between different biological replicates (**Figure 3D**). A gene ontology (GO) term analysis revealed that in iPSC-derived microglia lacking TREM2 “myeloid cell homeostasis”, “myeloid cell development”, and “myeloid progenitor cell differentiation”, and GO terms related to cell adhesion, motility, and migration as well as lipid metabolism and mitochondrial organization were enriched (**Figure S2**, red boxes). Together, this indicates that the transcriptional phenotypes of the TREM2 KO and wild type iPSC microglia are sufficiently different from each other in order to expect functional differences as well.

**Figure 4:**
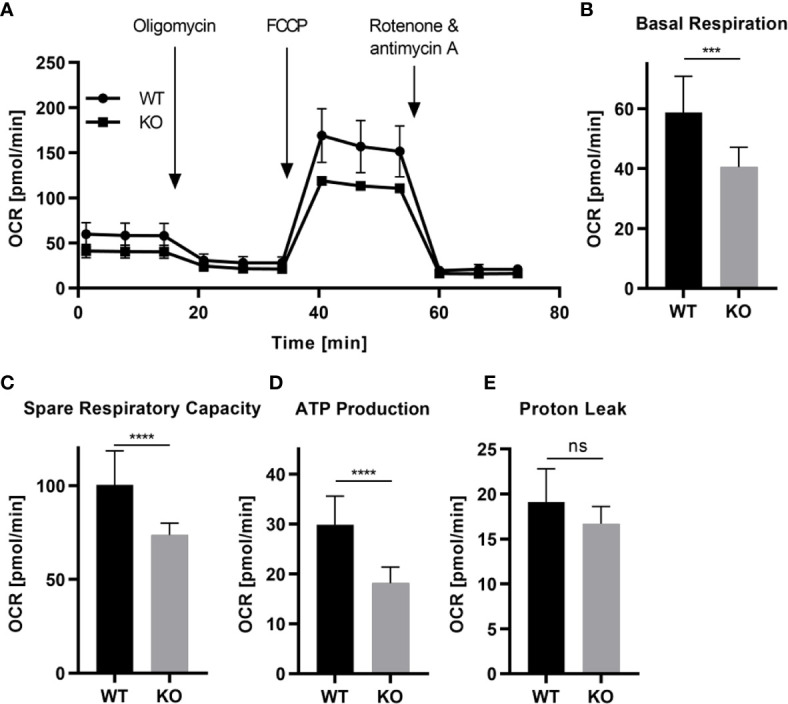
Differential mitochondrial respiratory activity in TREM2 KO versus wild type iPSC microglia. WT and TREM2 KO monoculture iPSC microglia-like cells were re-plated after 14 days of differentiation into fibronectin pre-coated seahorse assay plates **(A–E)**. **(A)** The baseline oxygen consumption rate (OCR) of WT and TREM2 KO cells was assessed, followed by the sequential injection of different indicated mitochondrial electron transport chain complex inhibitors (oligomycin and a combination of rotenone and antimycin A) and the mitochondrial uncoupler carbonyl cyanide-4-(trifluoromethoxy)phenylhydrazone (FCCP). OCR was measured three times initially and after each injection. **(B)** The basal respiration was calculated by subtracting the OCR of non-mitochondrial respiration (OCR after rotenone/antimycin A injection) from the baseline OCR. **(C)** The spare respiratory capacity results from the subtraction of the basal respiration from the maximal respiration [(OCR after FCCP injection) – (non-mitochondrial respiration)]. **(D)** ATP production is the OCR of the basal respiration with the proton leak subtracted. **(E)** The proton leak is the remaining OCR after injection with oligomycin without non-mitochondrial respiration. n=3; Data shown as mean with standard deviation. Statistical analysis was performed using t-test. (ns, p ≥ 0.05; ***p ≤ 0.001; ****p ≤ 0.0001).

To confirm some of the GO term associated functions and to explore feasibility of functional assays with microglia from our monoculture condition, we subsequently tested iPSC-derived microglia for their mitochondrial activity, cellular calcium responses and migratory capacity. First, we used Seahorse extracellular flux analysis to assess mitochondrial respiration. TREM2 KO microglia displayed a significantly lower basal mitochondrial respiration (**Figure 4A, B**), spare respiratory capacity (**Figure 4C**), and less ATP production (**Figure 4D**), while there was no significant difference detected in the proton leak (**Figure 4E**) when compared to isogenic wild type iPSC microglia, indicating reduced use of the respiratory chain for ATP production.

**Figure 5:**
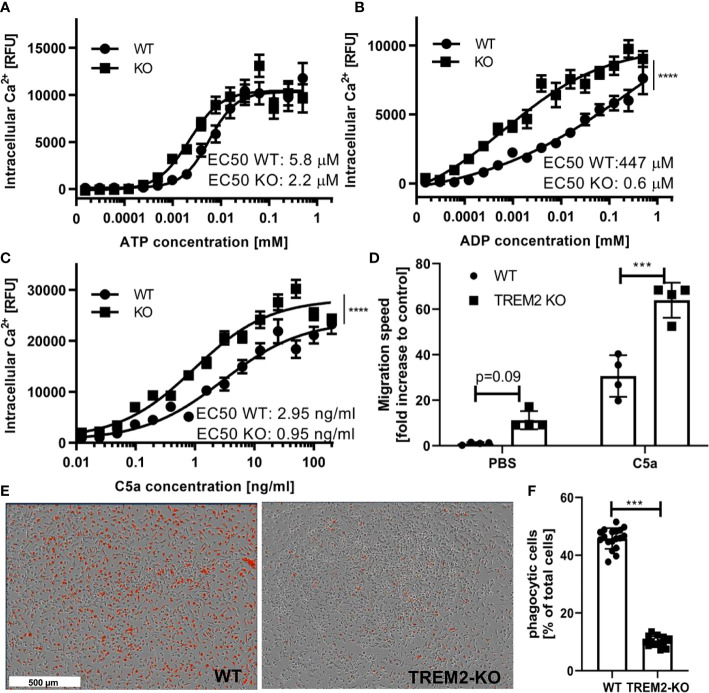
Functional differences in TREM2 KO versus wild type iPSC microglia. **(A–C)** Intracellular calcium kinetics following different stimuli. **(A)** Intracellular calcium levels upon stimulation with different concentrations of ATP. Maximum measured fluorescence is indicated as relative fluorescence units (RFU), which are baseline corrected (n = 3). Data shown as mean with standard error of the mean. Statistical analysis of two data sets was performed using a two-way ANOVA. **(B)** Intracellular calcium levels upon treatment with different concentrations of ADP. Maximum measured fluorescence is indicated as relative fluorescence units (RFU), which are baseline corrected (n = 3). Data shown as mean with standard error of the mean Statistical analysis of two data sets was performed using a two-way ANOVA(****p < 0.0001). **(C)** Intracellular calcium levels upon treatment of different concentrations of complement component 5a (C5a). Maximum measured fluorescence is indicated as relative fluorescence units (RFU), which are baseline corrected (n = 3). Data shown as mean with standard error of the mean. Statistical analysis of two data sets was performed using a two-way ANOVA (****p < 0.0001). **(D)** Migration speed of WT and TREM2 KO monoculture microglia in presence or absence of 1 ng/ml C5a as chemoattractant in the bottom compartment. Migration speed was calculated from the increase in iPSC microglia occupied area over time in the bottom compartment of the transwell and expressed relative to untreated WT control. Statistical analysis was performed using t-test for PBS and complement C5a condition, respectively (***p < 0.001). **(E)** Representative images of WT and TREM2 KO iPSC microglia incubated for 2 h with pH-rodo labeled Aß-coated beads. **(F)** Quantification of pHrodo positive microglia after 2 h incubation with pHrodo labeled Aß-coated beads. n = 3; Data shown as mean with standard deviation. Statistical analysis was performed using t-test (***p < 0.001).

Microglia continuously monitor their environment and can react to damage signals (e.g., ADP/ATP released by dying neurons, local activation of complement pathways in the aging brain and in neurodegeneration), i.e., by directed migration to the damage site (60). Many of the microglial receptors rely on changes of free intracellular calcium levels to mediate the internal signal transmission and integration. For instance, chemoattractants and damage-associated molecules signal through GPCR receptors thereby increasing intracellular calcium levels. To check for this functionality, we stimulated the cells with different concentrations of the typical chemoattractants and damage signals ADP, ATP and complement factor C5a (**Figures 5A–C**) and monitored the related intracellular calcium changes. The anaphylatoxin C5a is a well-described chemoattractant for innate immune cells that can be employed in cell culture (27). In an *in vivo* setting light induced microglial migration in the retina has been reported to be C5aR dependent (61) and in context of AD recent reports suggest a role of C5a in the regulation of microglial inflammatory response (62). ATP and its metabolites are also well-described chemoattractants for microglia through their plethora of receptors (63–65) Confirming the GO term result, TREM2 KO cells reacted already at lower concentrations and with a higher maximum to all three stimuli than their isogenic wild type iPSC microglia. This indicates a potentially more rapid signal integration in the TREM2 KO versus the wild type microglia. This is supported by a slightly elevated baseline migratory phenotype, and much more by the robust almost doubling of the migration speed of TREM2 KO versus wild type microglia upon stimulation by C5a (**Figure 5D**).

Phagocytosis and in particular uptake of Aβ—a major component of senile plaques in the AD brain—is a prominent function of microglia, which according to preclinical *in vitro* and *in vivo* models is reduced by TREM2 loss of function (39, 66–68). In line with literature, we observed a strongly diminished uptake of Aβ-coated beads by the TREM2 KO compared with the wild type microglia (**Figures 5E, F**) confirming a critical role of TREM2 in regulation of microglial phagocytosis. Together, these functional data confirm the DAM-like phenotype of our iPSC microglia; a phenotype which can be reversed by knocking out TREM2.

In conclusion, the overall results of our different approaches to generate iPSC microglia combined with gene expression and functional phenotypes confirm the feasibility to employ monoculture conditions to generate microglia-like cells, which express relevant functional gene sets including the key surface receptor TREM2. The ability to ablate specific genes such as TREM2 to reverse their DAM-like phenotype make these iPSC microglia a valuable tool for studying biological mechanisms relevant for AD and to perform compound screening and testing for drug discovery.

## Discussion

Studying microglia in humans and ultimately finding and testing novel therapeutic approaches targeting these cells remains a huge challenge. This is attributed to the heterogeneity of phenotypes microglia can acquire. For instance, proliferation, migration, phagocytosis, neurotrophic signaling, factor release for the modulation of immune function and blood-brain barrier integrity contribute to their high degree of plasticity (1–7, 69). This allows them to react to various cues and to switch between the phenotypes rapidly (8). Moreover, microglia are reported to have different regional abundance and activity states during aging, brain activity and neurodegenerative processes (58, 70–72). In addition, accessing microglia in the human brain is in the majority of cases only possible in postmortem tissue (73).

To overcome the need to study microglia in or isolated from humans, the field investigates rodent microglia *in situ* or as primary cells *in vitro* as a surrogate (38, 74, 75). Apart from the need to use large numbers of animals in order to isolate sufficient quantities of microglia, there are also many technical caveats when using rodent microglia; for instance batch-to-batch variations caused by the isolation process and a rapid switch of their previous gene expression pattern and phenotype (12, 13). Additionally, there are concerns about phenotypes induced by in-breeding, different mouse strains with different immune backgrounds and the often-limited translatability between mice and humans. This inter species translatability is of general concern for all animal models but especially for various receptors and ligands related to innate immunity and particularly microglia (74, 76).

Besides rodent models, microglia like cells have also been derived from human peripheral monocytes (77). These cells have been shown to resemble microglia specific gene signatures and functional properties. However, due to the difference in hematopoietic origin, these cells rather resemble infiltrating monocytes than brain resident microglia (77).

The iPSC technology together with the evolving understanding of microglial origin in mice and humans (21–23) allow now the robust generation of human iPSC-derived microglia-like cells (24–26, 78) in large amounts (25, 27).This provides the opportunity to employ human iPSC microglia for large-scale drug screening and for extensively studying biological mechanisms under more physiological and translational conditions. Additionally, iPSC based models provide the opportunity to study the effect of disease associated genes with isogenic mutation or knockout pairs (42, 43, 45). All of these protocols aim to follow the course of embryonic development and to recapitulate this *in vitro* as far as possible (24–27, 79). For the first part, the generation of myeloid progenitors *via* transcription factor MYB-independent primitive myelopoiesis, most protocols are broadly similar. However, the challenge for microglia generation as well as for the maintenance of primary microglia lies in the simulation of the correct neuronal microenvironment that determines the final differentiation and is essential for the maintenance of the phenotype (12). Several approaches exist for the *in vitro* simulation of this tissue niche, most of which are based on co-culture approaches with either neurons (25), astrocytes (42) or both. Other approaches use chemokines, morphogens and metabolites to differentiate the microglia-like cells in monoculture (17, 80)

Recently, we demonstrated the scalability of a protocol for the generation of myeloid precursors and primitive macrophages (27). The protocol is based on a publication by van Wilgenburg et al. (46) and cells generated with this differentiation protocol have already been used previously to obtain microglia like cells in co-cultures (2 and 3D) as well as in monoculture (25, 81). However, further optimization of the monoculture protocol seemed desirable. In our study, we aimed for such a reductionist approach of monoculture microglia and compared them on the morphological and gene expression level to the more elaborate co-culture model. Our observations on the transcriptional as well as on the phenotypic level correlate with the results reported from others (24, 25, 78)and extend these. The constitution of growth factors in our finally chosen microglia differentiation media is similar to the one published by McQuade and colleagues (78). Even though the two protocols differ in mesoderm induction and pre-Mac generation, resulting cells showed similar transcriptional and morphological profiles. However, a key advantage of the protocol shown here is the long lifetime of the myeloid factories, which allows continuous pre-Mac supply over a production period of 60–80 days (27).

We observed on the transcriptional level that monoculture-derived iPSC microglia showed an increase in genes related to the so-called DAM signature compared with neuronal co-culture derived microglia (**Figure 2**). The cell surface receptor TREM2 is part of the DAM signature (37, 38). It is an important molecule for microglia to interact with their environment and mutations in the gene for TREM2 are associated with neurodegenerative disorders including AD (9, 10, 31–36). Due to the importance of TREM2 for microglia and in drug discovery attempts for AD in general, we focused the characterization of our human iPSC microglia on TREM2 related functions and chose a TREM2 KO isogenic pair to further challenge the suitability of our monoculture model in cellular assays. First, we compared the isogenic controls on the transcriptional level and identified differences in genes associated with mitochondrial organization, cell motility and migration. Indeed, we could confirm differences in mitochondrial respiration by the Seahorse assay. Furthermore, we observed different responses in free intracellular calcium following exposure to well-established microglia chemoattractants, and pronounced differences in the migration towards one of these attractants (complement C5a) (**Figures 5A–D**), which is in line with observations by others (82). Furthermore, we demonstrated that the monocultured iPSC microglia are also suitable for phagocytosis assays (**Figures 5E, F**) and confirmed here observations published by others (39, 66–68). TREM2 signals *via* DAP12 and phosphorylation of SYK resulting in phosphorylation of the downstream kinases PI3K, PLCy and ERK (42, 67). These pathways have already been linked to alterations in cell proliferation, survival, metabolism, motility, and phagocytosis (42, 82–84). TREM2 KO has been shown to restore a homeostatic phenotype in AD and SOD models *in vivo* (82, 84). However, our observation that TREM2 KO cells show an increase in proliferative signatures and in motility was rather unexpected. One possible explanation could be that DAP12 may be stronger involved in the integration of other signaling cascades upon TREM2 loss. For the complete understanding of the pathway, we plan to explore the effect of loss of downstream targets on the cellular phenotypes in future studies.

Overall, our monoculture microglia model performed robustly in the applied functional cellular assays, pointing to its suitability to study cellular effects of TREM2 modulation. In a next step, our iPSC microglia model could be applied for cellular screening and profiling of various microglia modulatory pathways in the context of AD or other neurological diseases. Additionally, the established protocol to generate iPSC microglia now forms the basis to expand the single cell type culture with other cell types in order to generate multicellular spheroids and organoids. However, such complex cell models also come with greatly increased cultivation time, limited throughput and reduced number of available readouts (85) and more optimization will be required.

In this study, we have not addressed the suitability of the monoculture microglia system to investigate the effects of other disease-associated mutations or to model aspects of other diseases. However, the transcriptional data set in combination with the microglial module analysis provides a good basis to judge suitability of this model for other purposes. Using this as a starting point, one could test a variety of stimuli and see how they affect the expression in the different modules. Once we know what drives the differentiation of iPSC microglia towards a desired phenotype could enable the development of a toolbox for modeling key aspects of different microglial subtypes in monoculture.

In conclusion, the combined results of our experiments demonstrate that human iPSC microglia can be produced robustly in monoculture and our data confirm that these cells can be used in various microglia-relevant functional assays. Additionally, genetic ablation of TREM2 leads to the expected phenotypes validating these cells as a valuable tool for studying microglia-related biological mechanisms and to perform compound screening and testing for drug discovery.

## Data Availability Statement

The datasets presented in this study can be found in online repositories. The names of the repository and accession number can be found here: https://www.ncbi.nlm.nih.gov/geo/, GSE159108.

## Author Contributions

MB and SG conceived, designed, and supervised the study and SC and ME provided critical scientific input. MR, IP, ND, DR, and CS performed the cellular experiments. FK, MP, RS, and ME did the RNAseq analysis and evaluated the data. SG drafted the article and co-wrote the paper with MB. CP, SC, ML, MR, ME, and JZ revised the article critically. ML academically supervised MR’s master’s thesis, which is part of this manuscript. All authors contributed to the article and approved the submitted version.

## Funding

SG was supported by the Roche Postdoctoral Fellowship (RPF) program and IP by the Roche Internships for Scientific Exchange (RiSE) program.

## Conflict of Interest

During the course of this study, MR, IP, ND, CS, DR, RS, ME, JZ, CP, SG, and MB are or were full time employees or trainees at Roche and they may additionally hold Roche stock/stock options.

The remaining authors declare that the research was conducted in the absence of any commercial or financial relationships that could be construed as a potential conflict of interest.
