# Supplementary material for: Alzheimer’s Risk Gene TREM2 Determines Functional Properties of New Type of Human iPSC-Derived Microglia
Source: Front Immunol. 2021 Feb 3;11:617860. doi: 10.3389/fimmu.2020.617860 (PMC7887311; doi:10.3389/fimmu.2020.617860)
Supplement: Supplementary file 1 [file DataSheet_1.pdf]

Supplementary Information

Alzheimer's risk gene TREM2 determines functional properties of new type of human iPSC-derived microglia

Marvin Reich[1,2,7,$], Iñaki Paris[1,3,$], Martin Ebeling[4,] Nadine Dahm[5], Christophe Schweitzer[1], Dieter Reinhardt[1], Roland Schmucki[4], Megana Prasad[4], Fabian Köchl[4], Marcel Leist[2], Sally A. Cowley[6], Jitao David Zhang[4], Christoph Patsch[5,8], Simon Gutbier[5,*], and Markus Britschgi[1,*]

Supplementary Figures:

Fig.S.1 Culture condition optimization
Fig.S.2 GO-Term enrichment analysis

Supplementary Tabels:

Table.S.1 Media compositions
Table.S.2 Taq-man probes
Table.S.3 Microglia modules
Table.S.4 Antibodies

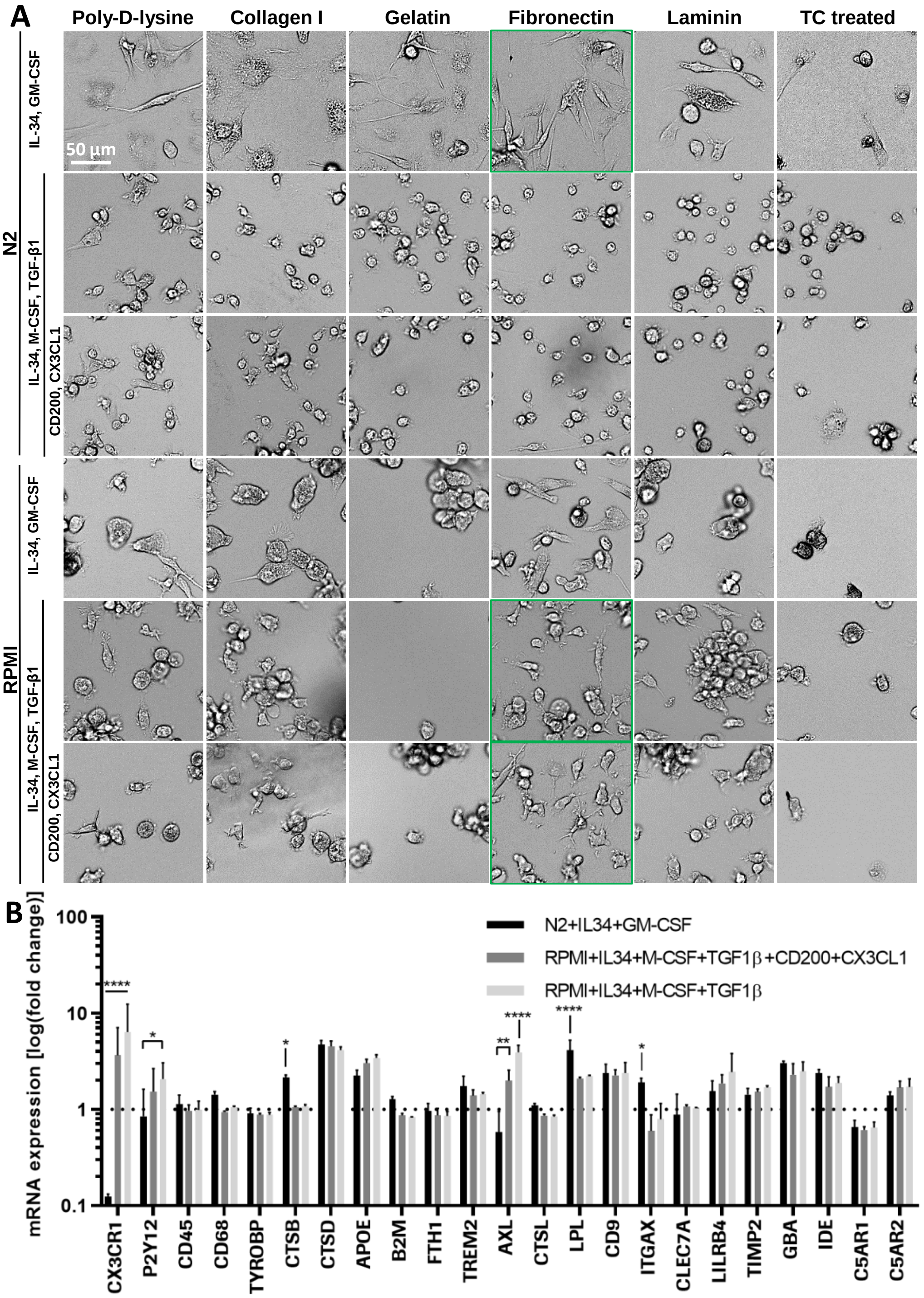

Fig.S.1 Culture condition optimization

(**A**) Representative bright-field images of the 36 conditions that were initially tested after 24 days of differentiation. Rows represent different media conditions, columns represent different coatings. For coating conditions multicoated microplates (Corning, Cat.No 3823) were used. Conditions chosen for further analysis are marked using green boxes. (**B**) The mRNA expression of microglial marker and DAM signature genes of microglia-like cells that were generated using three different protocols, after 24 days differentiation. Cumulated mRNA expression levels of three biological replicates (n = 9) are shown as log(10) fold changes compared to macrophage precursor cells.Data shown as mean with SD. Statistical analysis was performed using Tukey's multiple comparisons test (^{ns} $p \geq 0.05$, * $p < 0.05$, ** $p < 0.01$, *** $p < 0.001$, **** $p < 0.0001$).

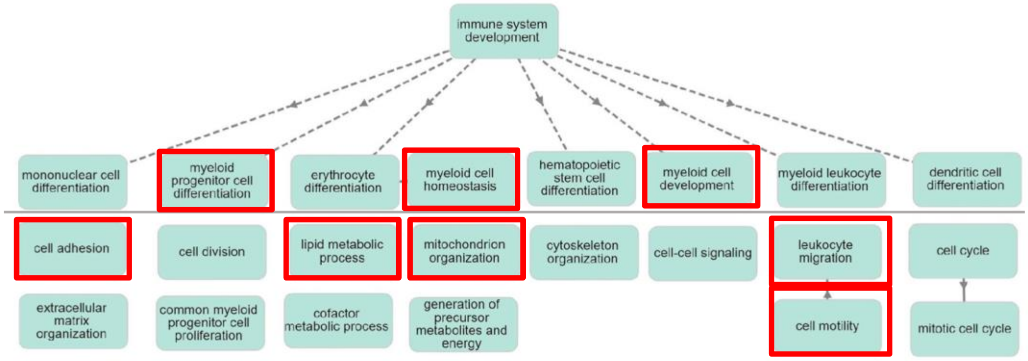

Fig.S.2 Outcome of a Gene Ontology analysis between WT and TREM2 KO iPSC microglia.

A Gene Ontology (OG) analysis using differentially expressed genes (arbitrary cut-off 4 reads per kilo base per million mapped reads, RPKM) revealed in TREM2 KO iPSC microglia enrichment of several GO terms related to cellular ontogeny and function compared with WT iSPC microglia (red boxes). Boxes in the upper row indicate GO terms connected with each other via the GO term ‘immune system development’ (dashed line arrows). The lower two rows indicate single enriched GO terms.

Media	Component	Concentration	Provider	Reference number
iPSC media	mTesR1		StemCell Technologies	85850
Neuron media	Advanced DMEM/F12 (with Glutamax)	1:1	Thermo Fisher Scientific	313328
	Neurobasalmedium	1:1	Thermo Fisher Scientific	21103049
	B27 supplement without vitamin A	1%	Thermo Fisher Scientific	12587010
	N2 supplement	1%	Thermo Fisher Scientific	1750204
	Beta-Mercaptoethanol	50 µg/ml	Thermo Fisher Scientific	31350-010
	rhBDNF	20 ng/ml	PeproTech	AF-450-02
	rhGDNF	10 ng/ml	PeproTech	K2315
	Aa2-P	100 µM	Sigma Aldrich	A8960
	cAMP	500 µM	BIOLOG Life Science	D009
EB formation media	mTesR1		StemCell Technologies	85850
	rhBMP4	50 ng/ml	biotechne	314-BP
	rhVEGF	50 ng/ml	biotechne	293-VE
	rhSCF	20 ng/ml	biotechne	235-SC
Factory media	X-VIVO 15		Lonza	BE02-053Q
	Penicillin/Streptomycin	10 U/ml	Thermo Fisher Scientific	15140-122
	GlutaMAX	2 mM	Thermo Fisher Scientific	35050-061
	Beta-Mercaptoethanol	50 µg/ml	Thermo Fisher Scientific	31350-010
	rhM-CSF	100 ng/ml	Miltenyi Biotech	130-096-493
	rhIL3	25 ng/ml	Miltenyi Biotech	130-095-069
N2 / Co-culture media	Advanced DMEM/F12		Thermo Fisher Scientific	12634-010
	N2 supplement	1%	Thermo Fisher Scientific	1750204
	Penicillin/Streptomycin	10 U/ml	Thermo Fisher Scientific	15140-122
	GlutaMAX	2 mM	Thermo Fisher Scientific	35050-061
	Beta-Mercaptoethanol	50 µg/ml	Thermo Fisher Scientific	31350-010
	rhIL34	100 ng/ml	Miltenyi Biotech	130-108-977
	rhGM-CSF	10 ng/ml	biotechne	215-GM
RPMI / Mono-culture media	RPMI 1640		Thermo Fisher Scientific	A10491-01
	Penicillin-Streptomycin	1%	Thermo Fisher Scientific	15140-122
	rhIL34	100 ng/ml	Miltenyi Biotech	130-108-977
	rhM-CSF	25 ng/ml	Miltenyi Biotech	130-096-493
	rhTGF-β1	50 ng/ml	PeproTech	100-21-50ug

Table.S.1 Media compositions

Gene	Dye	Taqman primer	Provider
APOE	FAM	Hs00171168_m1	Thermo Fisher Scientific
AXL	FAM	Hs01064444_m1	Thermo Fisher Scientific
B2M	FAM	Hs00187842_m1	Thermo Fisher Scientific
C5AR1	FAM	Hs00704891	Thermo Fisher Scientific
C5AR2	FAM	Hs01933768	Thermo Fisher Scientific
CD45	FAM	Hs04189704_m1	Thermo Fisher Scientific
CD68	FAM	Hs02836816_g1	Thermo Fisher Scientific
CD9	FAM	Hs01124022_m1	Thermo Fisher Scientific
CLEC7A	FAM	Hs01902549_s1	Thermo Fisher Scientific
CST7	FAM	Hs00175361_m1	Thermo Fisher Scientific
CTSB	FAM	Hs00947439_m1	Thermo Fisher Scientific
CTSD	FAM	Hs00157205_m1	Thermo Fisher Scientific
CTSL	FAM	Hs00964650_m1	Thermo Fisher Scientific
CX3CR1	FAM	Hs01922583_s1	Thermo Fisher Scientific
EMILIN2	FAM	Hs00230757_m1	Thermo Fisher Scientific
FTH1	FAM	Hs01694011_s1	Thermo Fisher Scientific
GBA	FAM	Hs00986836_g1	Thermo Fisher Scientific
GPR34	FAM	Hs00271105_s1	Thermo Fisher Scientific
IDE	FAM	Hs00610452_m1	Thermo Fisher Scientific
ITGAX	FAM	Hs00174217_m1	Thermo Fisher Scientific
LILRB4	FAM	Hs00359756_g1	Thermo Fisher Scientific
LPL	FAM	Hs00173425_m1	Thermo Fisher Scientific
MKI67	FAM	Hs04260396_g1	Thermo Fisher Scientific
OLFML3	FAM	Hs01113293_g1	Thermo Fisher Scientific
P2Y12	FAM	Hs01881698_s1	Thermo Fisher Scientific
P2Y13	FAM	Hs00256749_s1	Thermo Fisher Scientific
PPIA	VIC	Hs99999904_m1	Thermo Fisher Scientific
SLC2A5	FAM	Hs01086390_m1	Thermo Fisher Scientific
TIMP2	FAM	Hs00234278_m1	Thermo Fisher Scientific
TMEM119	FAM	Hs01938722_u1	Thermo Fisher Scientific
TREM2	FAM	Hs00219132_m1	Thermo Fisher Scientific
TYROBP	FAM	Hs00182426_m1	Thermo Fisher Scientific

Table.S.2 Taq-man probes

TREM2-independent	TREM2-dependent	Interferon	LPS	Macrophage	Microglia	Neurodegeneration	Monocyte	Proliferation
Keren-Shaul	Keren-Shaul	Friedman et al.	Friedman et al.	Friedman et al.	Friedman et al.	Friedman et al.	Friedman et al.	Friedman et al.
CTSB, CTSD,<br>APOE,<br>TYROBP,<br>GNAS,<br>FTH1,<br>B2M,	CST7<br>AXL<br>SPP1<br>CTSL<br>LPL<br>CCL6<br>CD9<br>CSF1	AKT3<br>IFI44<br>USP18<br>CMPK2<br>IFI16<br>IFIT2<br>IRF7<br>ISG20<br>IFIT1B<br>OAS2<br>OAS3<br>CHIC1<br>XAF1<br>RNF213<br>RTP4<br>ST8SIA1<br>STAT2<br>DHX58<br>ZBP1<br>NLRC5<br>HELZ2<br>OASL<br>RSAD2<br>ISG15	ADAM8<br>OPTN<br>IFITM3<br>OLFM4<br>CXCL13<br>IFITM2<br>MTHFD2<br>EHD1<br>LILRB4<br>CCR7<br>CD300LB<br>TMEM178A<br>CRIP1<br>CSF2RA<br>CD300LF<br>RASGEF1B<br>CYBB<br>SLFN5<br>MCEMP1<br>TMEM154<br>ALCAM<br>FGR<br>ATF5<br>PLXND1<br>SLC7A11<br>C19orf38<br>CCRN4L<br>CLIC4<br>GCNT2<br>NGFRAP1<br>CXCL3<br>GSN<br>PILRA<br>ERO1L<br>HINT1<br>IGF2R<br>IGFBP5<br>IL1RN<br>CXCL10<br>ITGA4<br>ITGA5<br>KCNA3<br>FAM132A<br>LMNA<br>ASNS<br>NFIL3<br>OLR1<br>PLAUR<br>FXYD5<br>SLAMF8<br>RBMS1<br>CCL5<br>SLC1A5<br>SLC7A1<br>TSPO<br>TKT<br>TNFAIP2<br>C3<br>IRG1<br>VCAM1<br>EZR<br>XDH<br>CACNA1S<br>PQLC1<br>RAB11FIP1<br>TUBB6<br>GPR65<br>TUBA1C<br>IQGAP1<br>MAPKAPK2<br>CD40<br>CD44<br>IKBKE	CLEC4M<br>CLEC10A<br>DAB2<br>F13A1<br>CD93<br>MRC1<br>PF4<br>CFP<br>SERPINB8<br>ARHGAP15<br>MS4A7<br>TGFBI<br>PLA2G7<br>CLEC6A	CSMD3<br>MED12L<br>CCR5<br>CST3<br>GPR155<br>CX3CR1<br>GCNT1<br>GPR34<br>GTF2H2<br>TMEM119<br>ARHGAP5<br>MFAP3<br>GOLM1<br>P2RY13<br>RAB39A<br>PMEPA1<br>SALL1<br>SELPLG<br>P2RY12<br>SPARC<br>TLR3<br>LRRC3<br>PLXDC2<br>CD164<br>GPR56<br>LRBA	SPEG<br>GPNMB<br>VAT1<br>POSTN<br>EGLN3<br>OSBPL8<br>ADSSL1<br>PLIN2<br>MFSD12<br>PLEKHH2<br>COX6A2<br>CPD<br>LRGUK<br>ASB10<br>PTCHD1<br>SRXN1<br>CSF1<br>CSPG4<br>CTSB<br>CTSV<br>CTSZ<br>S1PR1<br>PIANP<br>FABP5<br>ELL2<br>RFTN1<br>CADM1<br>ATP6V0D2<br>MAMDC2<br>RAI14<br>DKK2<br>SIGLEC8<br>GPR162<br>GNAS<br>GOT1<br>RASSF3<br>GAS2L3<br>CYP4V2<br>CD274<br>MDFIC<br>ANXA4<br>ANXA5<br>HIF1A<br>APBB2<br>RAB7B<br>HCAR2<br>ST8SIA6<br>TMEM202<br>APOC2<br>CD200R1L<br>IGF1<br>APOE<br>IL1R1<br>ITGAX<br>KCNJ2<br>KLRB1<br>LAG3<br>LGALS3<br>LGALS3BP<br>LOX<br>LPL<br>LY9<br>LYZ<br>MITF<br>MMP12<br>ASPH<br>MYO5A<br>ATP1A3<br>P2RX4<br>CHST11<br>CDK18<br>PDCD1<br>SERPINB6<br>SERPINE2<br>PLAU<br>FBLIM1<br>VPS13C<br>FAM46C<br>AXL<br>SULF2<br>ANKH<br>KIAA1217<br>PSEN2<br>FAM20C<br>ACTR3B<br>PTGER4<br>NCEH1<br>RAP2B<br>PLEKHA1<br>RXRG<br>CRLF2<br>XYLT1<br>INF2<br>CPEB1<br>CLEC7A<br>SOAT1<br>SPP1<br>ST14<br>TLR2<br>TYROBP<br>CACNA1A<br>CXCR4<br>GNPTAB<br>RNF128<br>PRR5L<br>COLEC12<br>OR5V1<br>CAPG<br>SLC41A2<br>BCAR3<br>CST7<br>BHLHE40<br>NRP1<br>SYNJ2<br>CH25H<br>SYNGR1<br>CD9<br>SOCS6<br>CD83<br>CD22<br>TTYH2<br>CHST2<br>CD34<br>CXCL14<br>CD68<br>CD72	LILRB3<br>CNN2<br>EMB<br>FLNA<br>SASH1<br>FPR2<br>PRDX5<br>CLEC4E<br>AOAH<br>HP<br>CLEC4D<br>ITGAL<br>LCN2<br>LTF<br>MGST1<br>MMP8<br>MSR1<br>MSRB1<br>TREM1<br>S100A6<br>S100A9<br>SELL<br>PLBD1<br>CAMP<br>EMILIN2<br>CCND2<br>GDA	SMC4<br>NDC80<br>CENPE<br>CENPF<br>HPSE<br>KIF2C<br>UBE2C<br>WDHD1<br>CDCA5<br>CKS1B<br>BTLA<br>DHFR<br>DNA2<br>DUT<br>CBX5<br>ASPM<br>CKAP2<br>GPR31<br>TRIM59<br>SLC43A3<br>ATAD2<br>UBE2T<br>RACGAP1<br>UHRF1<br>GPSM2<br>HELLS<br>HMMR<br>BIRC5<br>KIF11<br>LIG1<br>LMNB1<br>MCM2<br>MCM5<br>MCM6<br>MKI67<br>NUSAP1<br>PLK1<br>POLA1<br>POLE<br>POLE2<br>ANLN<br>NCAPG2<br>SPDL1<br>CDCA8<br>CEP55<br>MIS18BP1<br>MCM10<br>ASF1B<br>PBK<br>KIAA1524<br>RAD51<br>RRM2<br>FIGNL1<br>STIL<br>AURKA<br>TCF19<br>BUB1<br>TK1<br>TOP2A<br>UNG<br>WEE1<br>E2F8<br>CDT1<br>DIAPH3<br>CDCA3<br>NUF2<br>BRIP1<br>ARHGAP19<br>CYP4F2<br>CCNA2<br>TIMELESS<br>CCNE1<br>PRC1<br>CCNB2<br>CCNE2<br>AURKB<br>KIF23<br>KIAA0101<br>CDK1<br>MELK<br>GINS1

Table.S.3 Microglia modules

Antibody	Description	Used concentration	Provider	Reference number
Donkey anti-Goat	IgG, AF555	4 µg/ml	Invitrogen	A-21432
Donkey anti-Mouse	IgG, AF647	4 µg/ml	Invitrogen	A-31571
Donkey anti-Rabbit	IgG, AF488	4 µg/ml	Invitrogen	A-21206
GFAP	pAb rabbit	6.4 µg/ml	Agilent	Z0334
Iba1	pAb goat	2.5 µg/ml	abcam	ab5076
P2Y12	pAb rabbit	8 µg/ml	abcam	ab183066
TREM2	IgG1 mAb mouse	4 µg/ml	abcam	ab201621
TuJ1	IgG2A mAb mouse	2 µg/ml	BioLegend	801201/2

Table.S.4 Antibodies
